# BAY 1024767 blocks androgen receptor mutants found in castration-resistant prostate cancer patients

**DOI:** 10.18632/oncotarget.6864

**Published:** 2016-01-09

**Authors:** Tatsuo Sugawara, Pascale Lejeune, Silke Köhr, Roland Neuhaus, Hortensia Faus, Kathy A. Gelato, Matthias Busemann, Arwed Cleve, Ulrich Lücking, Franz von Nussbaum, Michael Brands, Dominik Mumberg, Klaus Jung, Carsten Stephan, Bernard Haendler

**Affiliations:** ^1^ Global Drug Discovery, Bayer Pharma AG, Berlin, Germany; ^2^ Berlin Institute of Urologic Research, Berlin, Germany; ^3^ Department of Urology, Charité University Hospital, Berlin, Germany

**Keywords:** prostate cancer, androgen receptor, anti-androgen resistance, mutant

## Abstract

Androgen receptor (AR) mutations arise in patients developing resistance to hormone deprivation therapies. Here we describe BAY 1024767, a thiohydantoin derivative with strong antagonistic activity against nine AR variants with mutations located in the AR ligand-binding domain (LBD), and against wild-type AR. Antagonism was maintained, though reduced, at increased androgen levels. Anti-tumor efficacy was evidenced *in vivo* in the KuCaP-1 prostate cancer model which bears the W741C bicalutamide resistance mutation and in the syngeneic prostate cancer rat model Dunning R3327-G. The prevalence of six selected AR mutations was determined in plasma DNA originating from 100 resistant patients and found to be at least 12%. Altogether the results show BAY 1024767 to be a strong antagonist for several AR mutants linked to therapy resistance, which opens the door for next-generation compounds that can benefit patients based on their mutation profile.

## INTRODUCTION

Treatments targeting aberrant androgen receptor (AR) signaling initially show impressive efficacy [1-5]. Unfortunately, therapy response usually only lasts for about 18 months, after which castration-resistant prostate cancer (CRPC) emerges. Several resistance mechanisms centering on the AR have been evidenced: rise in androgen supply by conversion of weak adrenal androgens into dihydrotestosterone (DHT) or by intratumoral *de novo* synthesis, increase of AR expression and gene copy number, mutations of AR leading to promiscuity and response to non-androgen ligands, and occurrence of splice variants with ligand-independent activity [6-9]. In addition, a number of genomic alterations may arise in the AR signaling pathway, which further underscores the essential role of the androgen axis in CRPC [10, 11].

Elevated androgen levels and AR overexpression can be addressed to some extent with AR antagonists possessing higher activity for the target, as exemplified by the recent approval of the second-generation AR antagonist enzalutamide [2, 4]. However, the development of antagonists addressing the most important AR mutants is compounded by the number of different variants identified, and the limited and sometimes conflicting information on their prevalence. Most AR mutations identified in CRPC are located in the ligand-binding domain (LBD) and alter the ligand-induced conformation of this region so that coactivator recruitment is still possible in the presence of antagonists, non-androgen steroids or weak adrenal androgens [12, 13]. In addition, different sets of downstream genes are controlled by AR mutants, implying that ligand- and mutation-selective conformations may take place [10, 14].

Conversion of antagonism to agonism in the presence of different AR mutants has been observed for approved AR antagonists. Cyproterone acetate, hydroxyflutamide and nilutamide stimulate AR T877A, the first AR mutation identified in prostate cancer [15]. Hydroxyflutamide and bicalutamide activate the AR V715M mutant [16]. Bicalutamide, but not hydroxyflutamide, becomes an agonist for the AR W741L and W741C mutants, due to the activation of an androgenic-like programme [10], further confirming that ligands with distinct chemical scaffolds have different allosteric effects on receptor conformation [17]. The E709Y mutant is strongly stimulated by bicalutamide, but less so by hydroxyflutamide or nilutamide [18]. TThe AR mutation F876L, which leads to activation by the recently approved enzalutamide and the related ARN-509 compound, has been identified by an *in vitro* selection procedure and an *in vivo* model selected for growth in the presence of the antagonist [19-21]. This mutation has already been detected in patients developing resistance to ARN-509 or enzalutamide [22, 23]. The AR H874Y mutant is stimulated by anti-androgens, adrenal androgens and non-androgen steroids, leading to enhanced coactivator recruitment [24, 25]. Several AR mutants not stimulated by anti-androgens but activated by various physiological steroids have also been found. For example, AR L701H is stimulated by glucocorticoids, whose novel interactions were revealed in modeling experiments [26]. Since this mutant shows little response to AR antagonists, the broad activation by non-androgen steroids is probably responsible for the tumor growth observed in prostate models bearing this mutation [27]. The mutations L701H, H874Y and T877A were also reported in patients with resistance to the C17,20 lyase inhibitor abiraterone. This may be due to previous treatment with AR antagonists or to co-medication with glucocorticoids, which activate AR mutants [20, 28].

In view of the persistent crucial role of the AR in most CRPC patients, there is a high need for novel antagonists addressing the adaptive mutations that emerge following anti-hormone therapy. Here we describe BAY 1024767, a novel and potent competitive antagonist of wild-type and mutated AR forms, and with potent *in vivo* efficacy. The prevalence of selected AR mutations was assessed in CRPC patients using the newly described BEAMing (Beads, Emulsions, Amplification, and Magnetics) technology to analyze circulating tumor DNA (ctDNA), and found to be at least 12%.

## RESULTS

### Identification of BAY 1024767

**Figure 1:**
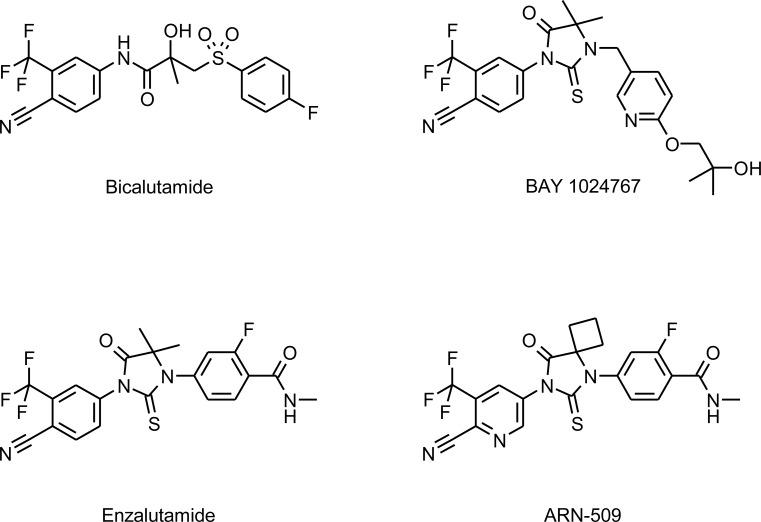
Chemical structures of the anti-androgens investigated

The synthesis of BAY 1024767 is described in patent WO 2011/029537 (A1) as example 10. The compound was discovered during a lead optimization project aiming at identifying highly potent AR antagonists with strong activity against wild-type and mutated AR forms. The crystal structure of AR mutant W741L bound to bicalutamide shows the influence of ligand shape on helix 12 conformation [29]. We developed novel antagonists that extend beyond the space occupied by the fluorophenyl ring of bicalutamide in order to displace helix 12 into an antagonist conformation, even in AR forms with an expanded ligand-binding pocket due to mutation. BAY 1024767 is a representative of the thiohydantoin type of anti-androgens substantiating this hypothesis (Figure 1).

### BAY 1024767 is a strong antagonist for wild-type and mutated AR

**Figure 2:**
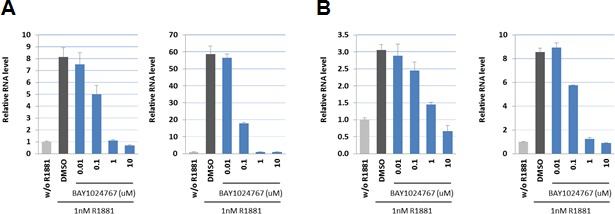
Down-regulation of androgen target genes in prostate cancer cells following BAY 1024767 treatment **A.** PSA (left) and FKBP5 (right) expression in VCaP cells. **B.** PSA (left) and FKBP5 (right) expression in LNCaP cells.

**Figure 3:**
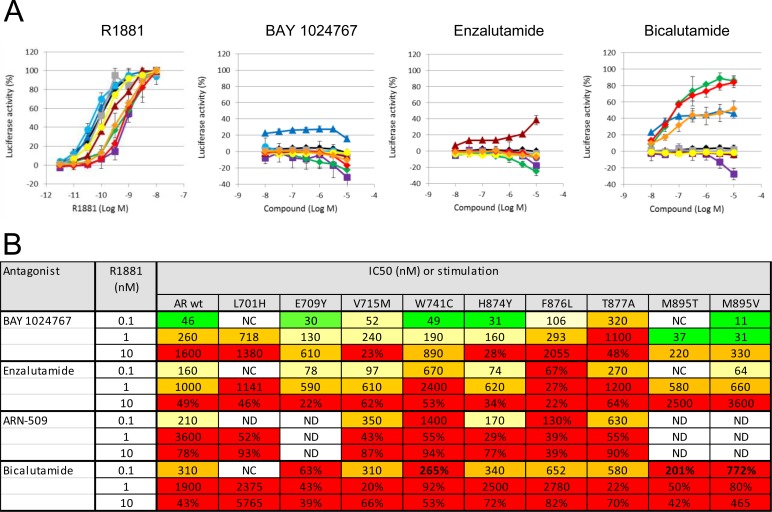
*In vitro* comparison of the activity of anti-androgens on AR wild-type and mutant forms **A.** Determination of agonistic effects. Transfected PC-3 cells were treated with the indicated concentrations of compounds, and luciferase activity of the reporter gene was measured 20 h later. Standard deviation from 2-4 independent experiments is shown. The curves show responses of different AR variants: wild-type (black), L701H (magenta), E709Y (dark blue), V715M (turquoise), W741C (green), H874Y (grey), F876L (burgundy red), T877A (yellow), M895T (red), M895V (orange). **B.** Table summarizing the antagonistic activities in cell-based transactivation assays. The indicated compounds were used to treat AR mutants in presence of different androgen concentrations. The calculated IC_50_ values are shown or, in case more than 20% activity was observed at the highest concentration tested, the residual activity remaining. Color code: green: ≤50 nM; yellow: 50-250 nM; orange: 250-1000 nM; red: ≥1000 nM or residual activity >20%. NC: not calculated due to insufficient activity of the mutated AR form. ND: not determined.

Binding of BAY 1024767 to human wild-type AR was determined in a competitive assay and found to be 450 nM. Target engagement was verified in VCaP (AR wild-type) and LNCaP (AR T877A mutant) cells treated with R1881 (Figure 2A and 2B). Comparable dose-dependent down-regulations of PSA and FKBP5 were observed following BAY 1024767 application. In light of AR alterations previously described in CRPC patients, the following nine mutations were chosen for testing: L701H, E709Y, V715M, W741C, H874Y, F876L, T877A, M895T and M895V. PC-3 cells were transfected with plasmids harboring each mutation and an MMTV-controlled luciferase reporter. The levels of transfected AR mutants were comparable and in the same range as endogenous AR in LNCaP cells, when measured by ELISA (Supplementary Figure S1). The synthetic androgen R1881 stimulated AR wild-type and most mutants similarly, with however weaker responses of the L701H, W741C, M895T and M895V forms (Figure 3A). No dose-dependent agonistic activity was observed with BAY 1024767 for any of the mutants tested (Figure 3A). Enzalutamide did not stimulate the mutants tested, with the remarkable exception of F876L, reaching nearly 40% of the R1881 activity at the highest concentration used (Figure 3A), as previously described [19, 21, 23]. In sharp contrast, bicalutamide did not activate F876L, but did stimulate several of the other mutants already at low concentrations, reaching almost the activity seen with 1 nM R1881 at elevated levels in the case of W741C and M895T. Less but still significant stimulation was observed for E709Y and M895V. No stimulatory effect of bicalutamide was detected for the 5 other mutants tested (Figure 3A). Next, the antagonistic activity of the compounds was compared using different R1881 concentrations for stimulation (Figure 3B). BAY 1024767 was a strong antagonist for all mutants tested and superior to the other anti-androgens examined, with the exception of the T877A mutant (Figure 3B). Enzalutamide was a strong antagonist at low R1881 concentration, however lost most of its activity for both wild-type and mutated AR when increasing the treatment to 10 nM R1881 (Figure 3B). Bicalutamide displayed a comparatively weak antagonism for AR wild-type in presence of 0.1 nM R1881, which was rapidly lost when increasing the stimulation to 1 or 10 nM R1881. Reduced or no antagonism was observed for all mutants tested especially at low R1881 concentrations. The novel clinical AR antagonist ARN-509 was weaker than enzalutamide but slightly stronger than bicalutamide. When comparing the overall resistance of the AR mutants tested, it is clear that T877A is the most challenging to address. Altogether BAY 1024767 was the only anti-androgen tested which displayed antagonism for all AR mutants and retained activity at elevated androgen stimulation.

### BAY 1024767 promotes AR nuclear localization

**Figure 4:**
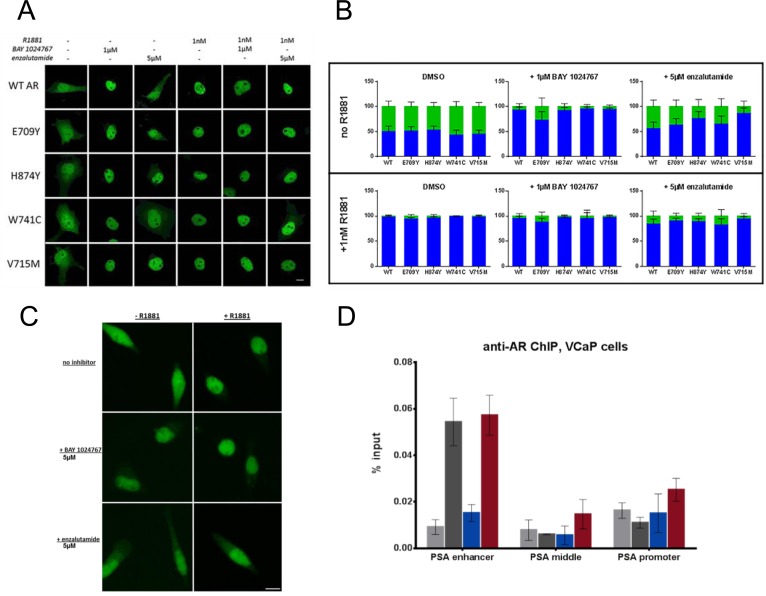
Effects of BAY 1024767 and enzalutamide on AR sub-cellular localization and chromatin binding **A.** Transfected COS-7 cells were treated with the indicated compounds for 4 h and then analyzed by laser scanning microscopy, using a FITC-labeled AR antibody. The scale bar indicates 10 μm. **B.** AR nuclear and cytoplasmic distribution of 50 cells per treatment were quantitated using ImageJ. Results are presented as percentage of fluorescence signal overlapping with the DAPI nuclear region (blue bars) or in the cytoplasm (green bars). Error bars indicate the standard deviation. **C.** Cellular localization of endogenous AR in VCaP cells was analyzed using the antibody in part A. Representative images for each treatment are shown. The scale bar indicates 10 μm. **D.** ChIP was performed on VCaP cells following compound treatment for 6 h with 1 nM R1881 with DMSO (dark grey), 1 nM R1881 with 5 μM BAY 1024767 (blue), or 1 nM R1881 with 5 μM enzalutamide (red). No treatment controls (ethanol with DMSO) are shown in light grey. RT-PCR was performed with primers specific for the PSA enhancer or middle region, or the promoter. Error bars indicate the standard deviation from triplicate experiments.

Immunofluorescence studies using a FITC-labeled AR antibody were performed in transfected COS-7 cells treated with compounds for 4 h. AR wild-type and the mutants E709Y, V715M, W741C and H874Y were distributed in the cytoplasm in the absence of ligand but concentrated in the nucleus following R1881 treatment (Figure 4A and 4B). BAY 1024767 promoted nuclear localization of AR wild-type and mutants in the absence of R1881 with little residual cytoplasmic signal remaining upon antagonistic treatment alone, and did not prevent nuclear translocation in the presence of R1881. The results were similar following treatment with enzalutamide, however the translocation effects were slightly less pronounced, possibly due to weaker target engagement. Similar results were observed with endogenous AR in VCaP cells (Figure 4C).

### BAY 1024767 releases AR from the PSA gene enhancer region

Given that BAY 1024767 antagonized R1881-stimulated gene transcription but was not capable of preventing the translocation of AR into the nucleus, its mode of action was investigated at the chromatin-binding level in VCaP cells. AR binding to PSA gene regulatory elements is well characterized [30]. Chromatin immunoprecipitation (ChIP) showed AR binding after agonist treatment to be mostly at the PSA enhancer region, but not at the middle region or at the promoter. The strong AR binding to the PSA enhancer was entirely prevented by BAY 1024767, but not by enzalutamide (Figure 4D). No significant changes were observed at the middle or promoter region of the PSA gene, however a slight increase of bound AR was observed after enzalutamide treatment, compared to the control or to BAY 1024767-treated samples.

### *In vitro* anti-proliferative activity of BAY 1024767

**Figure 5:**
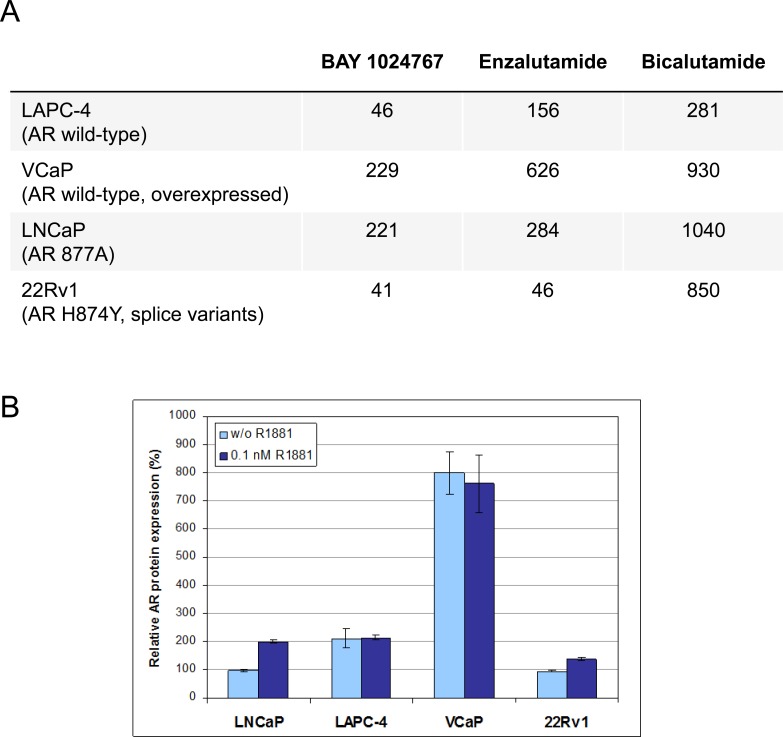
Anti-proliferative activity of anti-androgens in prostate cancer cell lines **A.** GI_50_ values are indicated in nM. **B.** Determination of AR protein levels by ELISA. The amount of AR measured in untreated LNCaP cells (light blue bars) was set to 100%, and levels after 0.1nM R1881 treatment (dark blue bars) are shown for each cell line.

The efficacy of BAY 1024767 was determined in different prostate cancer cell lines representative of clinically relevant resistance mechanisms (Figure 5A). In LAPC-4 cells which express AR wild-type, BAY 1024767 was superior to enzalutamide and to bicalutamide. In VCaP cells which contain 4- to 5-fold more AR protein than other prostate cancer cell lines (Figure 5B), BAY 1024767 was more effective at inhibiting cell proliferation than bicalutamide and enzalutamide. In LNCaP and 22Rv1 cells, containing mutated and/or splice variants of the AR, BAY 1024767 and enzalutamide had a comparable anti-proliferative effect, much stronger than that of bicalutamide. No anti-proliferative activity was observed when treating the AR-negative PC-3 cell line (Supplementary Figure S2).

### Pharmacokinetic properties of BAY 1024767

The pharmacokinetic properties of BAY 1024767 are summarized in Supplementary Table S1. Low hepatic clearance was measured in liver microsomes from different species including humans. The cell permeability was high and no efflux was observed. *In vivo* pharmacokinetic studies in male NMRI mice revealed a low clearance and a high oral bioavailability, thereby supporting oral administration as the application route for *in vivo* studies. After multiple oral doses of 50 mg/kg every second day to male SCID mice, unbound plasma concentrations of BAY 1024767 remained over the unbound anti-proliferative GI_50_ values determined in VCaP cells for the entire dosing interval. This was also true for bicalutamide dosed at 108 mg/kg daily and enzalutamide dosed at 160 mg/kg daily (Supplementary Figure S3).

### *In vivo* efficacy of BAY 1024767

**Figure 6:**
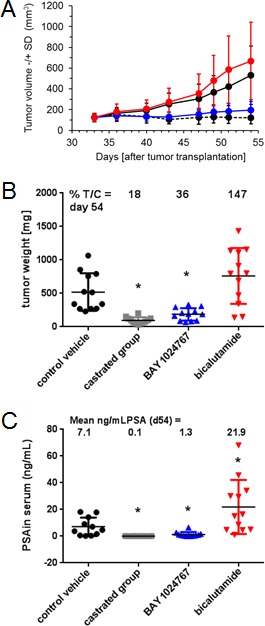
Comparison of the anti-tumor efficacy of BAY 1024767 and bicalutamide in the patient-derived KuCaP-1 model which contains a mutated AR form **A.** Tumor volume −/+SD changes during the course of the experiment. Control (black, solid line), control castrated (black, dashed line), BAY 1024767 (50 mg/kg, q2dx11; blue) and bicalutamide (60 mg/kg, qdx21; red) are shown with standard deviation at each time point measured. **B.** Tumor weight at the end of the experiment. *p < 0.05 *vs*. control vehicle group, one-way ANOVA on ranks, Dunn´s method. **C.** Determination of serum PSA levels. *p < 0.05 *vs*. control vehicle group using Mann-Whitney rank sum test.

The KuCaP-1 model harbors the AR W741C mutation [31]. Mice with subcutaneous implantation of KuCAP-1 fragments were treated orally once every other day with BAY 1024767 or daily with bicalutamide, once the tumor reached an approximate mean of 100 mm^3^. BAY 1024767 applied at 50 mg/kg/administration (maximal tolerated dose) showed strong efficacy, and tumor volume remained almost constant, near the levels of the group treated by castration (Figure 6A). In sharp contrast, bicalutamide (60 mg/kg/day) did not affect tumor growth. Tumor weight measured at the end of the study confirmed the efficacy of BAY 1024767 (36% T/C on day 54 post-tumor implantation, significant difference (*p* < 0.05) *versus* control group), whereas bicalutamide tended to stimulate tumor growth (147% T/C) (Figure 6B), as previously described [31]. Treatments were well tolerated with mean body weight losses similar (bicalutamide) or inferior (BAY 1024767) to that of the control group, and mostly related to the tumor burden (Supplementary Figure S4). BAY 1024767 strongly reduced serum PSA levels (1.3 ng/ml, compared to 7.1 ng/ml in the control group (Figure 6C)). In contrast, bicalutamide treatment led to a 3-fold increase of serum PSA (21.3 ng/ml) when compared to the non-treated group. In a different study using the same tumor model, enzalutamide was compared to bicalutamide (Supplementary Figure S5). Enzalutamide, applied orally daily at 100 mg/kg was inactive (with 100% T/C calculated on tumor weights on day 52 post-tumor implantation). As previously, bicalutamide (100 mg/kg, orally, daily) was inactive with 112% T/C.

**Figure 7:**
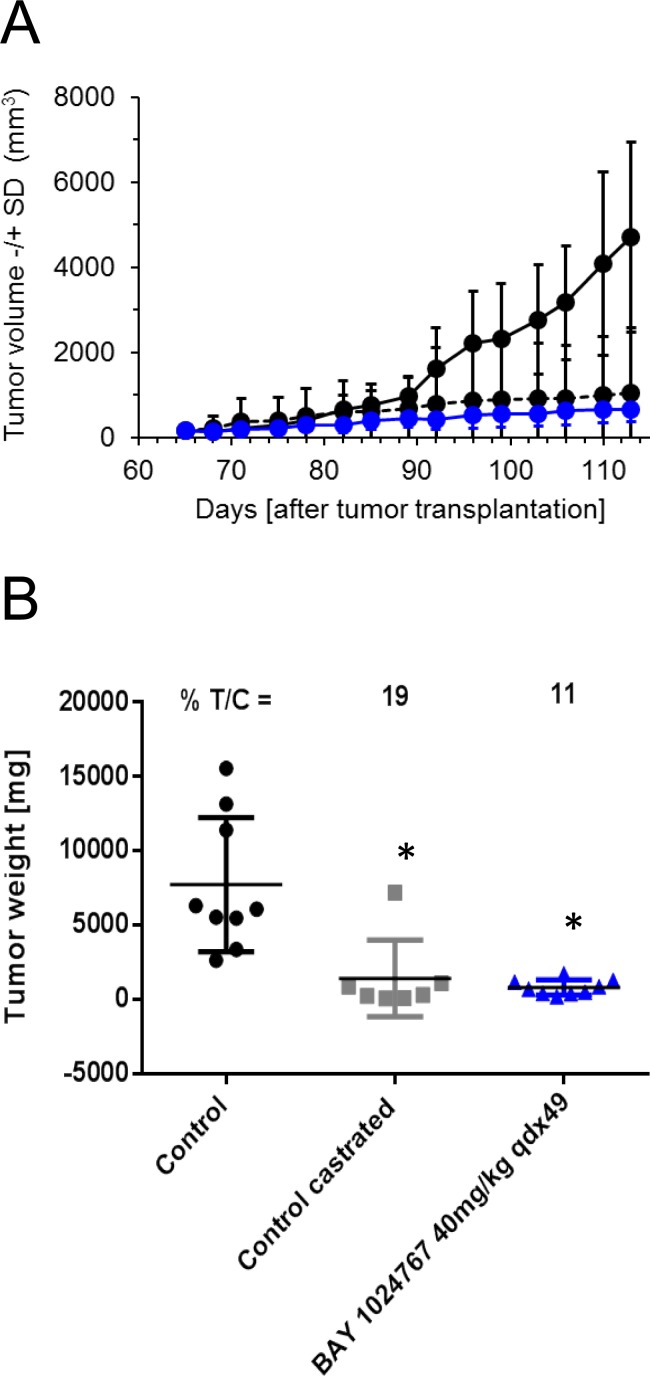
Anti-tumor efficacy of BAY 1024767 in the syngeneic Dunning rat R3327-G model **A.** Tumor volume −/+ SD measured during the course of the experiment for control (black, solid line), control castrated (black, dashed line) and BAY 1024767 (40 mg/kg, qdx49; blue) are shown with the standard deviation of each time point measured. **B.** Tumor weight at the end of the experiment. *p < 0.05 *vs*. control vehicle group, one-way ANOVA on ranks, Dunn´s method.

We additionally determined the *in vivo* efficacy of BAY 1024767 in the syngeneic Dunning rat R3327-G prostate cancer model [32]. Rats with subcutaneous implantation of tumor pieces were treated daily with oral doses of 40 mg/kg BAY 1024767, once the tumor reached 50 mm^3^. Comparison of tumor volumes showed a significant anti-tumor efficacy of BAY 1024767 (Figure 7A). This was also reflected at the level of tumor weights measured at the end of the study (11% T/C at day 113 post-tumor implantation, significant difference (*p* < 0.05) *versus* vehicle group (Figure 7B)). In addition, strong inhibition of seminal vesicle weight was observed following treatment with BAY 1024767, comparable to that seen in the castrated group (*p* < 0.05 *versus* vehicle group, data not shown). No significant body weight loss was observed, showing that treatment with BAY 1024767 at an efficacious dose was well tolerated in rats as well.

### Prevalence of AR mutations in CRPC patients

In order to determine the relevance of the AR mutations analyzed in this study in the clinical setting, we collected plasma samples from 100 patients who had received anti-hormone therapy and developed resistance. These samples were used for ctDNA purification and subsequent analysis with the very sensitive BEAMing technology [33]. Probes addressing six selected AR mutations were designed and in each case only one possible nucleotide change was selected, based on previously published information (Supplementary Table S2) [34]. The results (Table 1) reveal that 12 out of 100 CRPC patients had at least one out of the six investigated AR mutations (= 12%, 95% Jeffreys confidence interval: 6.73%, 19.43%). No correlation was observed with the Gleason score or the total PSA value. In two cases, two mutations were present in the same patient, indicating tumor heterogeneity. Interestingly, the T877A mutation was observed most frequently, accounting for five out of 14 cases in twelve different patients. Mutations at positions V715, W741 and H874 were each observed three times. The mutation frequency varied significantly among samples and was in several cases below 0.1%. In patient 5 however, a very high frequency of the T877A mutation was observed. These ratios should however be interpreted with caution since the amount of contaminating lymphocyte DNA may vary in each sample. Nonetheless the very high frequency and the fact that it was found in four additional patients underscore the importance of the T877A mutation in the acquisition of resistance to hormonal treatment in prostate cancer. It should also be noted that all the mutation frequencies determined may represent an underestimation since other DNA point mutations leading to the same amino acid exchange are possible but would not have been detected by the primers used in this study.

**Table 1:** Frequency of AR mutations in plasma DNA from CRPC patients as determined using the BEAMing technology

Patient identifier	Gleason score	PSA (ng/ml)	Bone mets	Other mets	Time to resistance (years)	G2507A V715M	G2585T W741C	G2982T H874Y	A2991G T877A	A3045G M895V	T3046C M895T
2	9	30.3	Yes	No	3	wt	0.19%	wt	wt	wt	wt
5	7	117.0	Yes	Yes	6	wt	wt	wt	12.43%	wt	wt
7	8	68.2	Yes	No	2	0.22%	wt	wt	wt	wt	wt
12	9	151.9	Yes	No	10	0.08%	wt	wt	wt	wt	wt
16	6	25.0	Yes	No	18	wt	wt	wt	0.03%	wt	wt
40	8	261.0	Yes	No	11	wt	wt	0.25%	wt	wt	wt
41	7	56.5	Yes	Yes	9	wt	wt	wt	0.21%	wt	wt
66	9	51.9	Yes	Yes	5	0.02%	wt	wt	0.06%	wt	wt
70	9	170.9	Yes	Yes	2	wt	wt	1.34%	0.04%	wt	wt
83	9	78.0	Yes	No	1	wt	0.10%	wt	wt	wt	wt
85	10	129.8	Yes	Yes	2	wt	0.43%	wt	wt	wt	wt
89	7	282.4	Yes	No	1	wt	Wt	0.03%	wt	wt	wt

PSA: prostate-specific antigen; mets: metastases.

## DISCUSSION

Resistance mechanisms described in CRPC mostly concern the AR pathway so that the identification of novel anti-androgens with improved properties remains an essential research focus in this field [3-5]. Indeed several new AR antagonists have moved to the clinical stage in recent years, raising the hope that new therapy options will be available soon [35-37].

BAY 1024767 is a novel competitive AR antagonist that exhibits strong activity against AR wild-type and mutated forms found in therapy-resistant patients, keeps antagonistic activity with increased androgen stimulation and in prostate cancer models with elevated AR protein levels. It also shows anti-proliferative activity in a model expressing splice variants. Its potent antagonism when androgen or AR levels are high is most probably due to strong target engagement whereas its activity towards mutants may be linked to its extended structure that presumably displaces helix 12 towards an antagonist position. The efficacy of BAY 1024767 was evidenced in various *in vitro* transactivation assays using mutated AR forms. *In vivo*, we observed very strong activity in the KuCaP-1 model which bears the comparatively frequent W741C AR mutation. Importantly, the compound also strongly impaired AR binding to the regulatory enhancer region of the PSA gene while reducing PSA serum levels in tumor-bearing mice, which is routinely used as a pharmacodynamic marker in the clinic. The efficacy of BAY 1024767 was further evidenced in the syngeneic Dunning R3327-G rat model with high AR expression where a significant anti-tumor effect was observed. In addition, a dramatic reduction of seminal vesicle weight, comparable to that seen in castrated animals, was evidenced.

AR mutations identified in CRPC patients usually do not merely lead to loss of interaction with antagonists but rather represent gain-of-function alterations. The mutations identified in ctDNA are likely to represent drivers of acquired resistance and among them T877A was the most frequent. This was unexpected since this mutation was originally identified in a patient treated with hydroxyflutamide [38], which is now rarely used for treatment. The recent finding that progesterone stimulates this mutant and is synthesized by the tumor may explain why this position is a mutation hotspot [39, 40]. Bicalutamide exhibits antagonism for the T877A mutant, which can be explained by the different positioning of AR LBD helix 12 [29]. Nonetheless, we found bicalutamide to be a weaker antagonist for this mutant in comparison to AR wild-type following stimulation with 0.1 nM androgen, and to lose most of its antagonistic activity at higher androgen levels. Indeed, loss of antagonism for the AR T877A mutant was seen for all compounds tested, but BAY 1024767 and enzalutamide maintained the best profile. This was mirrored in the proliferation assays with the LNCaP cell line, which harbors the T877A mutation, where BAY 1024767 and enzalutamide were also more efficacious than bicalutamide. The data underline that this position is key in the switch between an inactive and active AR conformation necessary for recruitment of co-activators and binding to target genes.

The occurrence of AR splice variants in CRPC patients has been reported [9, 41]. Many have lost their LBD and can therefore not be directly addressed by a competitive antagonist. However splice variants may form heterodimers with full-length AR wild-type, which are still blocked by anti-androgens [42, 43], and it will be interesting to find out whether this is also the case for heterodimers formed with AR LBD mutants.

The 12% frequency rate measured in our study for AR mutations is probably an underestimation. Firstly, despite the very high sensitivity of the BEAMing method, some of the interrogated mutations may have gone undetected, as the plasma preparations used in our study had not been optimized for enrichment of ctDNA. Secondly, for each selected position only one specific primer sequence was used to analyze the plasma DNA samples even though additional options existed in most cases. Thirdly, additional AR LBD mutations not addressed in this study have been identified recently, including F876L which was detected in 3 out of 27 patients treated with the novel AR antagonist ARN-509 [23]. In a very recent study on ctDNA from 62 patients progressing on enzalutamide or abiraterone, the AR mutation rate was found to be 18% by targeted next-generation sequencing of exon 8, which covers the C-terminal part of the LBD [22]. As in our study, the H874Y and T877A mutations were found several times. In addition, the M895V substitution was detected once, underlining the importance of having a compound such as BAY 1024767 for blockade of this AR mutant which was originally identified in a primary prostate tumor [44]. A sequencing approach was also used in another study focusing on circulating tumor cells (CTCs) from CRPC patients [45]. Here, 35 CTC samples were analyzed and 19 AR missense mutations identified in 15 patients. These mutations include several positions that we detected, and again M895V. Such unbiased sequencing approaches have the potential to identify all AR mutations arising in refractory prostate cancer patients provided the complete coding region is analyzed, but are unlikely to be used routinely in the near future. Also, they necessitate the isolation of CTCs, which is more cumbersome than purification of plasma DNA, and cannot be used in patients with low or no CTC counts.

Precise determination of the respective prevalence of individual AR mutations and their variation along treatment duration should now be more readily feasible using blood-based assays and the BEAMing technology described here, compared to analysis of CTCs which is not yet routinely established in the clinic, or of tumor biopsies which is challenging, especially in the case of metastases. Such approaches will deliver essential information for guiding the future treatment of CRPC, which is multifocal in nature and will necessitate bespoke drugs addressing both wild-type and mutated AR, while keeping efficacy in the presence of high androgen levels and at elevated AR expression.

In summary, we have identified BAY 1024767, a novel AR antagonist which addresses three essential mechanisms responsible for CRPC. This may offer new therapy options by overcoming and/or delaying resistance, either by treating patients who do not respond or who are stimulated by approved anti-hormonal agents, or by sequential use of AR antagonists with different profiles.

## MATERIALS AND METHODS

### Cell culture and materials

All anti-androgen compounds were synthesized in-house and are shown in Figure 1. The cell lines were obtained from ATCC (Manassas, VA, USA) or DSMZ (Braunschweig, Germany) and authenticated at the DSMZ by short tandem repeat DNA typing. The KuCaP-1 patient-derived model [46] was from Prof. O. Ogawa, University of Kyoto, Japan. The Dunning R3327-G rat prostatic tumor [47] and the AR expression plasmid [48] were described before. Site-directed mutagenesis was performed using the appropriate primer pairs and the QuickChange II XL Site-Directed Mutagenesis kit (Agilent Technologies, Santa Clara, CA, USA).

### Binding and transient transfection assays

Competitive binding to recombinant human AR was determined in presence of 5 nM tritiated R1881. PC-3 transfection and determination of luciferase activity were performed as before [49]. The test compounds were added with 0.1% ethanol (agonistic mode) or the indicated R1881 concentrations (antagonistic mode). The average value of six wells treated in parallel was taken. The experiments were repeated at least twice independently. The NR Sandwich AR ELISA 49696 (Active Motif, Carlsbad, CA, USA) was used to determine endogenous AR levels and compare the levels of expressed AR protein in transfected PC-3 cells.

### mRNA expression assays

VCaP or LNCaP cells were seeded into 6-well plates in RPMI 1640 medium without phenol red, supplemented with 10% charcoal-stripped fetal calf serum (cFCS) and 2 mM stable glutamine, and maintained for 2 days prior to treatment. R1881 (or ethanol control) was added to cells at 1 nM end concentration simultaneously with DMSO, or the AR antagonist BAY 1024767 at the indicated concentrations. After 24 h treatment, total RNA was extracted using the RNeasy Mini Kit (Qiagen, Hilden, Germany) and reverse-transcription was performed using the SuperScript™ III First-Strand Synthesis SuperMix for qRT-PCR (Invitrogen, Carlsbad, Germany). Transcript levels of AR target genes were determined by real-time PCR using the following assays (Applied Biosystems, Foster City, CA, USA): PSA Hs02576345_m1 and FKBP5 Hs00188025_m1. Human PPIA (Cyclophilin A) 4326316E was used as an internal control.

### Immunofluorescence

COS-7 cells were grown to 60% confluence and then incubated in phenol red-free DMEM medium containing 10% cFCS. After two days, cells were transfected with AR expression plasmids (1.25 ng) using Lipofectamine 2000 (Invitrogen) for 20 h, then were treated for 4 h with 1 nM R1881 or ethanol along with DMSO, 1 μM BAY 1024767 or 5 μM enzalutamide.

VCaP cells were grown to 30% confluence and then incubated in phenol red-free DMEM medium containing carbon-filtered FCS (DMEM-cFCS) for 3 days. The cells were treated for 6 h with 1 nM R1881 or ethanol along with DMSO, 5 μM BAY 1024767 or 5 μM enzalutamide.

For staining, culture medium was removed and the cells were washed three times with PBS. The cells were then fixed in 4% paraformaldehyde in PBS for 15 min at room temperature, washed, permeabilized with PBS containing 0.5% Triton X-100 for 10 min, washed again, treated with blocking solution (1% BSA, 0.1% Tween-20 in PBS) for 30 min, and incubated overnight at 37°C in blocking solution in the presence of anti-AR-FITC antibody (sc-815-FITC; Santa Cruz Biotechnology, Dallas, TX, USA). The cells were then washed, stained with 300 nM DAPI in PBS for 5 min, and sealed with a coverslip. Cells were imaged on an LSM700 confocal laser scanning microscope (Zeiss, Göttingen, Germany), using a 25X objective. Quantification of COS-7 cells was performed on 25 cells in two replicates using ImageJ software.

### Chromatin immunoprecipitation

VCaP cells were grown in 10 cm plates to 80% confluence. One plate of cells was used per immunoprecipitation. The cells were maintained in DMEM-cFCS for 3 days prior to treatment. R1881 (or ethanol control) was added to cells at 1 nM simultaneously with DMSO, or the AR antagonists BAY 1024767 or enzalutamide at 5 μM. After 6 h treatment, the cells were cross-linked in 1% formaldehyde in PBS for 8 min, quenched in 125 mM glycine, washed 3 times with PBS, and frozen. After cell lysis (5 mM PIPES pH 8, 85 mM KCl, 0.5 % Nonidet P40) and nuclei isolation using RIPA buffer, chromatin was sheared to 100-2000 base pair-long fragments using a S220 ultrasonicator (Covaris, Woburn, MA, USA).

Immunoprecipitations were performed using the IPstar device (Diagenode, Liège, Belgium) with 20 μL of magnetic Protein A bead suspension. 2 μg of antibodies anti-AR (sc-815-x; Santa Cruz) and rabbit IgG (Sigma-Aldrich, Taufkirchen, Germany) were used per immunoprecipitation. The buffers used were: bead wash (10 mM Tris-HCl pH 8, 1 mM EDTA, 0.02 % Tween-20), wash 1 (20 mM Tris-HCl pH 8, 2 mM EDTA, 150 mM NaCl, 1 % Triton X-100, 0.1 % SDS), wash 2 (20 mM Tris-HCl pH 8, 2 mM EDTA, 500 mM NaCl, 1 % Triton X-100, 0.1 % SDS), wash 3 (10 mM Tris-HCl pH 8, 1 mM EDTA, 250 mM LiCl, 1 % Nonidet P40, 1 % sodium deoxycholate) and elute (0.1 M NaHCO3, 1 % SDS).

Reverse cross-linking of immunoprecipitation elutions and inputs was done for 16 h at 65°C, with 160 μM NaCl final concentration. The samples were treated with RNaseA and Proteinase K, and were purified using the Qiagen PCR purification kit before analysis by qPCR.

The primers used were for the prostate specific antigen (PSA) gene [30, 50]: promoter forward CCT AGA TGA AGT CTC CAT GAG CTA CA; promoter reverse GGG AGG GAG AGC TAG CAC TTG; enhancer forward GCC TGG ATC TGA GAG AGA TAT CAT C; enhancer reverse ACA CCT TTT TTT TCT GGA TTG; middle forward CTG TGC TTG GAG TTT ACC TGA; middle reverse GCA GAG GTT GCA GTG AGC C.

### Cell proliferation assays

All assays were performed in 96-well microtiter plates. Cells were grown in RPMI-1640 media without phenol red and supplemented with 10% cFCS and 2 mM L-glutamine. LAPC-4 cells were plated at 4,000 cells/well, VCaP cells at 16,000 cells/well, LNCaP cells at 2,000 cells/well, 22Rv1 cells at 4,000 cells/well. After 1 day, the cells were treated with R1881 (0.1 nM final concentration, except for LAPC-4 where the final concentration was 1 nM) and compound (day 0). Cell number was determined by Alamar Blue staining (2 h). Fluorescence was measured in a Victor3 device (PerkinElmer, Waltham, MA, USA) with the excitation filter set at 530 nm and the emission filter set at 590 nm. C0 was defined as the signal measured at day 7 for cells treated only with R1881 and CI was defined as the signal measured at day 7 for cells grown without R1881 or compound.

### Metabolic stability *in vitro*

The metabolic stability of BAY 1024767 was determined by incubating the compound with suspensions of liver microsomes from different species at a concentration of 1 μM. The intrinsic clearance was calculated from the half-life of the compound. Together with additional parameters like microsomal protein content, species-specific liver weight and liver blood flow, the hepatic *in vivo* blood clearance (CL_H_) was calculated.

### Caco-2 permeation assay

Caco-2 cells were seeded on 24-well insert plates, 0.4 μm pore size, and grown for 15 days. The test compound was dissolved in DMSO and added either to the apical (A) or basolateral (B) compartment at a final concentration of 2 μM. Before and after 2 h incubation, samples were taken from both compartments and analyzed by LC/MS/MS. The apparent permeability (Papp) was determined for each direction and the efflux ratio was calculated as Papp B-A/Papp A-B.

### *In vivo* pharmacokinetics in mice

BAY 1024767 was administered to 6-7 week-old male NMRI mice in the tail vein by a single dose of 0.5 mg/kg and intragastral by a single dose of 1 mg/kg formulated in PEG400/water (60/40). Three mice were sacrificed at different time points after dosing, and blood (for heparinized plasma) was sampled from the *vena cava*. Analysis of the samples was performed by LC/MS/MS and pharmacokinetic parameters were calculated by non-compartmental analysis.

### *In vivo* efficacy studies

Animal experiments were approved by the relevant regulatory agency of the federal state of Berlin (Landesamt für Gesundheit und Soziales Berlin). Animals were housed according to institutional guidelines with access to food (pelleted diet) and water *ad libitum*. The KuCaP-1 patient-derived tumor model was maintained by serial passage in SCID/CB17 male mice. The presence of the W741C mutation was confirmed by DNA sequencing (not shown). For the efficacy studies, 11-12 week-old SCID/CB17 male mice (Charles River Laboratories, Erkrath, Germany) were inoculated subcutaneously with tumor fragments into the right flank. Tumors were allowed to grow and mice were randomly assigned to control or treatment groups with a mean tumor volume of 100 mm^3^ (*n* = 10-12 mice/group). Mice received vehicle, BAY 1024767 (50 mg/kg every 2 days [Q2D]), enzalutamide (100 mg/kg once daily [QD]) or bicalutamide (60 or 100 mg/kg once daily [QD]) orally for 18-21 days, depending on the study. The doses used were either the maximal tolerated dose or the dose reaching maximal exposure in mice. Compounds were solubilized in NMP/PEG300 1/9 (v/v). An additional group included mice castrated at the start of the study that received vehicle. Mouse body weight was determined at least twice a week. Tumors were measured twice a week using a caliper (tumor volume (mm^3^) = [length (mm) x width^2^ (mm^2^)]/2). Tumor/control ratio (T/C) % was calculated as [mean tumor weight in the treated group/mean tumor weight in the control vehicle group]×100). Efficacy was evaluated according to the National Cancer Institute standards (%T/C ≤ 42% = active). Statistical analysis was performed using a one-way analysis of variance (ANOVA) on ranks using Dunn's method. The Quantikine^®^ Human Kallikrein 3/PSA Immunoassay (R&D Systems, Minneapolis, MN, USA) was used to measure mouse serum PSA levels at the end of the study. Statistical analysis was performed using Mann-Whitney rank sum test.

The Dunning R3327-G tumor [51] was maintained by serial *in vivo* passages. For the efficacy experiment, tumor tissue pieces of 8 mm^3^ were inoculated subcutaneously (flank region) in 12-13 week-old male Copenhagen rats (COP/CrCrl strain, Charles River Laboratories). Treatment was started once the tumor reached 50 mm^2^ and rats were randomly assigned to control (*n* = 9), castrated (*n* = 10) or treatment group (*n* = 10). In the treatment group 40 mg/kg of BAY 1024767 was given orally once a day (p.o., QD). The vehicle was NMP/PEG 1/9 (v/v). Rat body weight was measured at least twice a week. Tumor volume and weight were determined as above.

### AR mutation frequency

All patients received written information about the planned AR mutation analysis performed on serum samples and signed a document indicating their informed consent. Blood plasma was collected and prepared from CRPC patients under standardized clinical laboratory practices. A total of 100 patients of which 98 had received at least two different hormonal treatments and with a median time to CRPC status of 8 years (range 1-20 years) were analyzed. Seventy-seven of these patients had bone metastases, 59 had visceral metastases and 49 had both. Blood plasma (2 ml) obtained from each patient was collected in EDTA tubes, mixed gently and centrifuged at 4°C for 10 min at 3400 g. It was then transferred by pipetting into 15 ml conical tubes which were centrifuged at 4°C for 15 min at 1500 g. The cell-free plasma was then transferred into storage tubes and kept at −80°C. The BEAMing experiments [52] were performed on cell-free plasma DNA by Sysmex Inostics (Hamburg, Germany). For the analysis, cell-free plasma DNA was first pre-amplified in a multiplex PCR reaction, amplified with nested primers and normalized. Emulsion PCR was then performed on the surface of magnetic beads in water-in-oil emulsions. Following destruction of the emulsion structure, the uncovered DNA fragments were hybridized to fluorescent probes specific to the mutations. These labeled beads were then quantified by flow cytometry. Statistical analysis was performed as described previously [53].

## SUPPLEMENTARY MATERIAL FIGURES AND TABLES


