# Supplementary material for: BAY 1024767 blocks androgen receptor mutants found in castration-resistant prostate cancer patients
Source: Oncotarget. 2016 Jan 9;7(5):6015–28. doi: 10.18632/oncotarget.6864 (PMC4868737; doi:10.18632/oncotarget.6864)
Supplement: Supplementary file 1 [file oncotarget-07-6015-s001.pdf]

BAY 1024767 blocks androgen receptor mutants found in castration-resistant prostate cancer patients

Supplementary Material

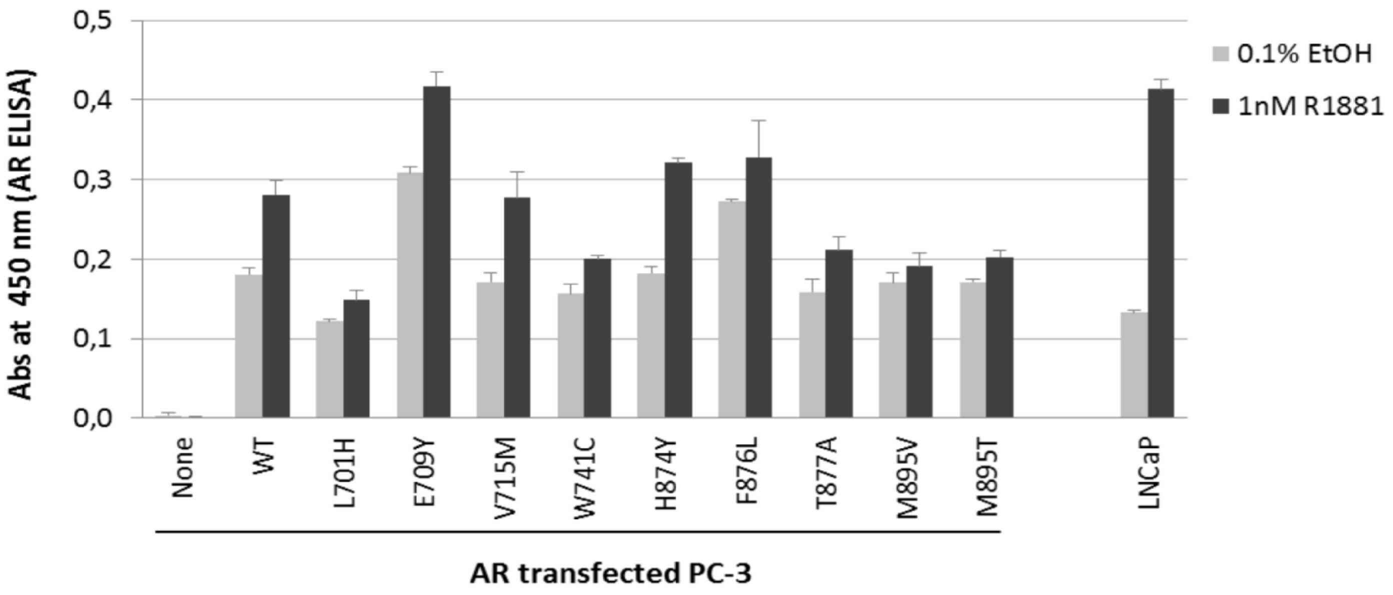

Figure S1.

Comparison of transfected AR protein levels in PC-3 cells and endogenous AR in LNCaP cells. Plasmids encoding the indicated AR mutants were used for transfection. AR was measured with the NR Sandwich AR ELISA kit 49696 from Active Motif in cells treated or not with R1881. SD for duplicate experiments are shown.

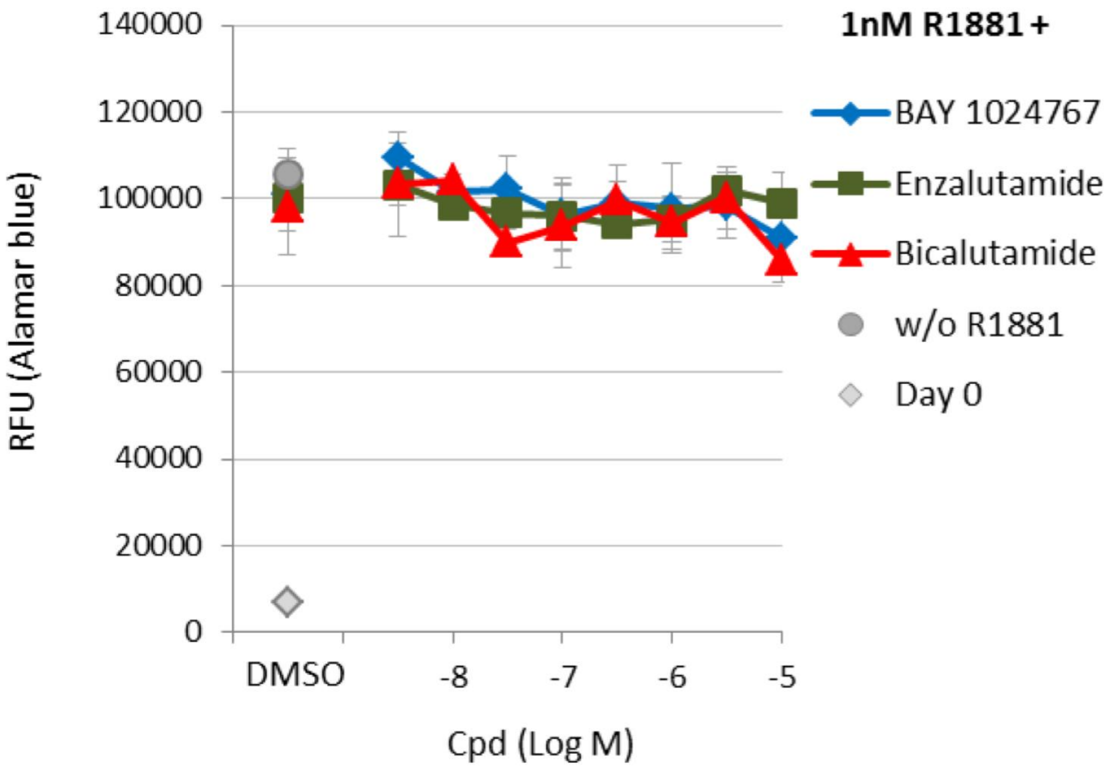

Figure S2.

Treatment of the AR-negative prostate cancer cell line PC-3 cells with anti-androgens. The indicated concentrations of compounds were added to PC-3 cells seeded in 96-well plates for 4 days. Cell viability was determined using the Alamar blue assay.

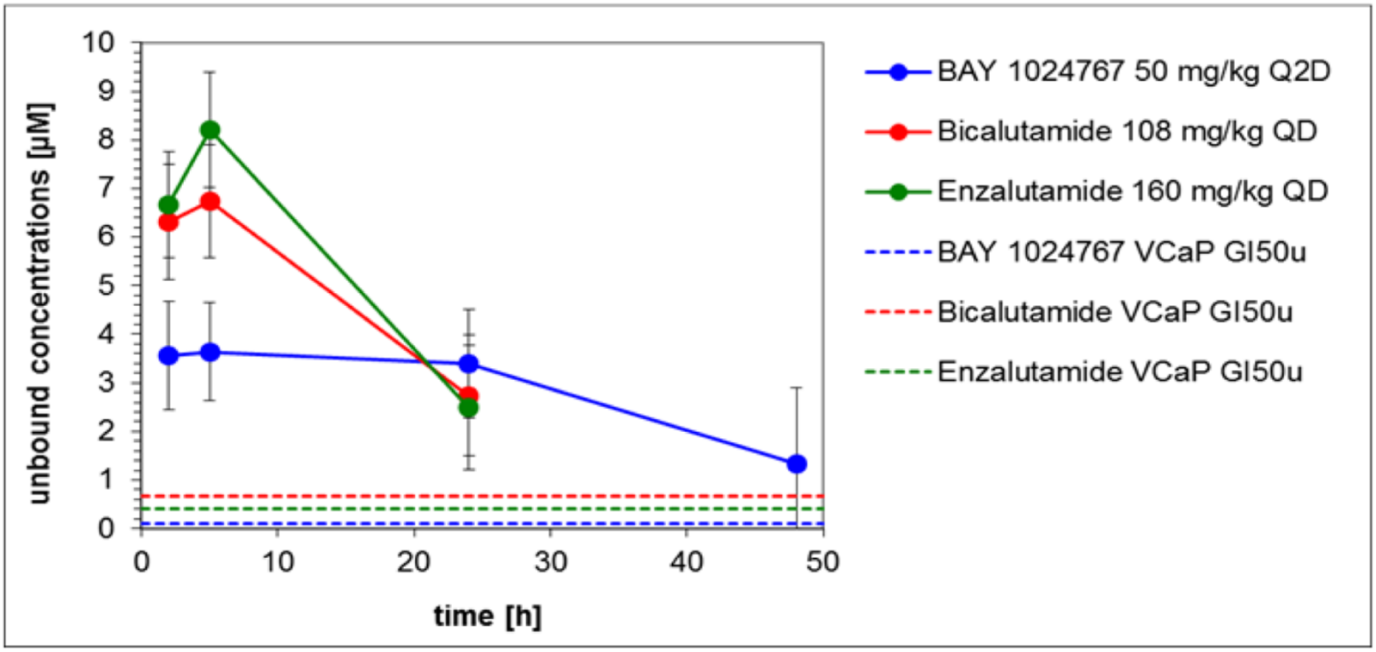

Figure S3.

Pharmacokinetic profiles in mouse. Unbound plasma concentrations of BAY 1024767, bicalutamide and enzalutamide after administration of multiple oral doses of 50, 108 and 160 mg/kg, respectively to male SCID mice. BAY 1024767 was dosed every other day (Q2D), bicalutamide and enzalutamide were dosed daily (QD). Dashed lines indicate *in vitro* unbound anti-proliferative GI_{50} values measured in VCaP cells. Unbound concentrations -/+ SD for 2-3 animals are shown.

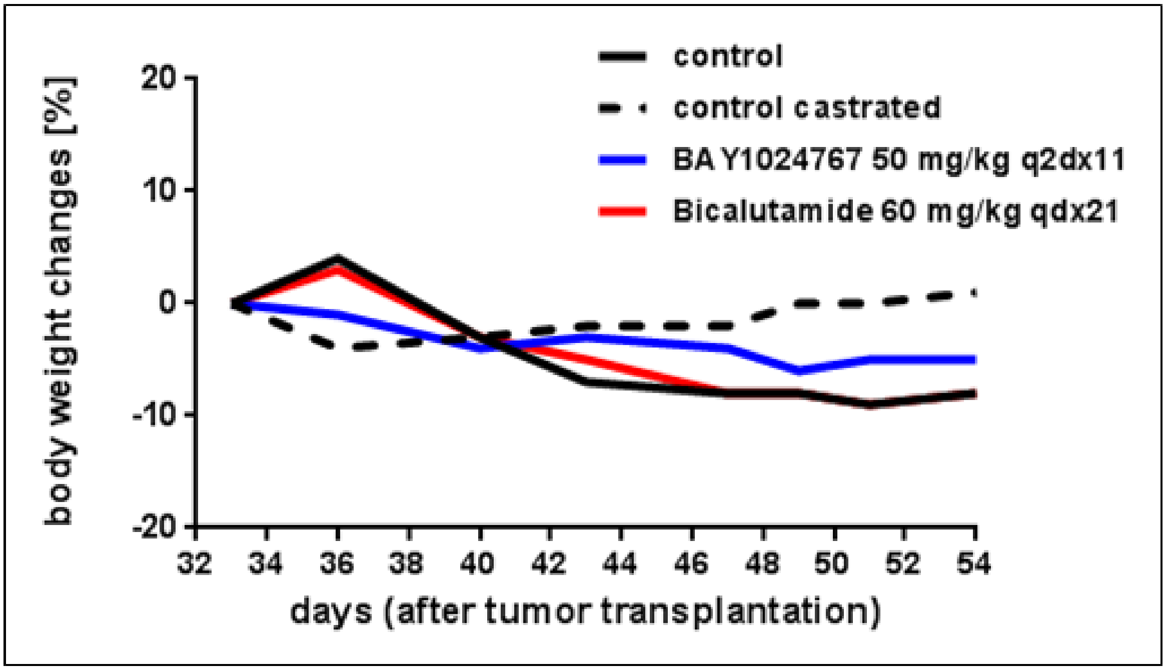

Figure S4. Body weight changes in mice bearing the KuCaP-1 model and treated with BAY 1024767 or bicalutamide.

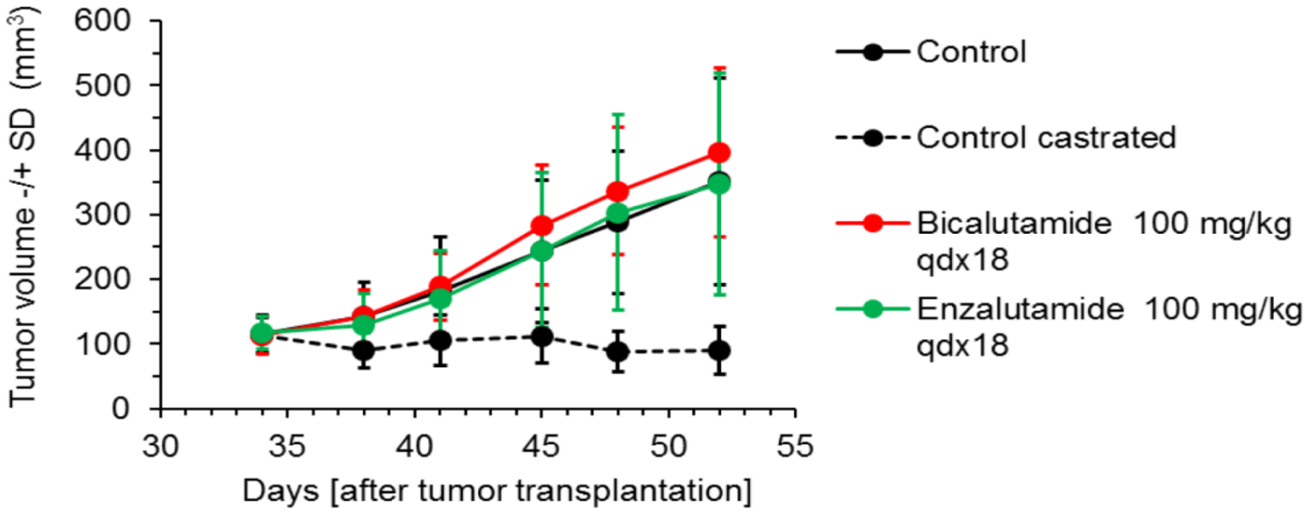

Figure S5. Comparison of the anti-tumor efficacy of enzalutamide and bicalutamide in the patient-derived KuCaP-1 model. Tumor volumes -/+ SD measured during the course of the experiment are shown. No statistical difference between the control goup and enzalutamide- or bicalutamide-treated groups.

Table S1. Pharmacokinetic properties of BAY 1024767.

***In vitro* hepatic clearances determined with liver microsomes from mouse (m), rat (r), dog (d), monkey (mk) and human (h); permeability/transport in Caco-2 cells; *in vivo* PK parameters in mouse: blood clearance (CL_B), volume of distribution (V_{ss}) and oral bioavailability (F).**

Metabolic stability microsomes	CL_H (L/h/kg)	m, r, d, mk, h<br>0.19, 0.28, 0.27, 0.70, 0.35
Permeability Caco-2	Papp A-B (nm/s)<br>Efflux ratio	183<br>0.61
Mouse pharmacokinetics	CL_B (L/h/kg)<br>V_{SS} (L/kg)<br>F (%)	0.06<br>0.61<br>99

Table S2. DNA mutations analyzed with the BEAMing technology. The location in the AR gene as well as the interrogated DNA mutation and the corresponding amino acid exchange are listed.

Gene	Exon	Nucleotide			Amino acid change
		Position	Change	Report	
AR	4	2507	G>A	G2507A	V715M
AR	5	2585	G>T	G2585T	W741C
AR	8	2982	C>T	C2982T	H874Y
AR	8	2991	A>G	A2991G	T877A
AR	8	3045	A>G	A3045G	M895V
AR	8	3046	T>C	T3046C	M895T
